# Time Course Analysis of Genome-Wide Identification of Mutations Induced by and Genes Expressed in Response to Carbon Ion Beam Irradiation in Rice (*Oryza sativa* L.)

**DOI:** 10.3390/genes12091391

**Published:** 2021-09-09

**Authors:** Jian Zhang, Ziai Peng, Qiling Liu, Guili Yang, Libin Zhou, Wenjian Li, Hui Wang, Zhiqiang Chen, Tao Guo

**Affiliations:** 1 National Engineering Research Center of Plant Space Breeding, South China Agricultural University, Guangzhou 510642, China; jian_zhang@stu.scau.edu.cn (J.Z.); pengziai123@163.com (Z.P.); liu17853516178@163.com (Q.L.); yanggl@scau.edu.cn (G.Y.); wanghui@scau.edu.cn (H.W.); chenlin@scau.edu.cn (Z.C.); 2 Institute of Modern Physics, Chinese Academy of Sciences, Lanzhou 730000, China; libinzhou@impcas.ac.cn (L.Z.); wjli@impcas.ac.cn (W.L.)

**Keywords:** carbon ion beam (CIB), whole-genome sequencing (WGS), RNA-seq, mutation characteristics, initial DNA damage, DNA repair-related genes

## Abstract

Heavy-ion irradiation is a powerful mutagen and is widely used for mutation breeding. In this study, using whole-genome sequencing (WGS) and RNA sequencing (RNA-seq) techniques, we comprehensively characterized these dynamic changes caused by mutations at three time points (48, 96, and 144 h after irradiation) and the expression profiles of rice seeds irradiated with C ions at two doses. Subsequent WGS analysis revealed that more mutations were detected in response to 40 Gy carbon ion beam (CIB) irradiation than 80 Gy of CIB irradiation at the initial stage (48 h post-irradiation). In the mutants generated from both irradiation doses, single-base substitutions (SBSs) were the most frequent type of mutation induced by CIB irradiation. Among the mutations, the predominant ones were C:T and A:G transitions. CIB irradiation also induced many short InDel mutations. RNA-seq analysis at the three time points showed that the number of differentially expressed genes (DEGs) was highest at 48 h post-irradiation. Kyoto Encyclopedia of Genes and Genomes (KEGG) pathway analysis of the DEGs showed that the “replication and repair” pathway was enriched specifically 48 h post-irradiation. These results indicate that the DNA damage response (DDR) and the mechanism of DNA repair tend to quickly start within the initial stage (48 h) after irradiation.

## 1. Introduction

Spontaneous mutations have been the primary drivers of evolution ever since life on Earth began [1]. Almost all life relies on spontaneous mutations to create new genetic variation to adapt to the Earth’s environment, but these mutations occur very infrequently (7 × 10^−9^ in *Arabidopsis thaliana*) [2]. However, the ability to artificially induce mutations in species has changed this situation. In recent decades, several mutagens, both physical and chemical ones, such as X-rays [3], γ rays (GRs) [4], fast neutrons [5], ethylmethanesulfonate (EMS) [6], and heavy-ion beams (HIBs) [7], have been used for generating genetic variability and for plant mutation breeding. Among them, HIB irradiation has been recognized as a powerful mutagen and has been extensively utilized in plant breeding because it can generate new cultivars with desired traits without affecting other traits [8,9]. Based on many physical characteristics, such as high linear energy transfer (LET), mass and energy deposition, large damaged sections, and strong penetration ability, compared with conventional low-LET irradiation (such as X-rays and GRs), HIB irradiation easily produces single-strand breaks (SSBs) and causes double-strand breaks (DSBs), cluster damage, and other forms of DNA damage that are difficult to repair.

Next-generation sequencing (NGS) technology is a powerful technique for single-nucleotide polymorphism (SNP) detection and for assessing the effects of induced mutations. With the development of sequencing technology, DNA sequencing costs have drastically decreased. An increasing number of researchers have applied NGS technology to characterize the molecular nature of induced mutations [10,11,12]. Hirano et al. [13] comprehensively characterized induced mutations in the genome of *A. thaliana* plants irradiated with Ar or Fe ions and they subsequently used the information to survey the mutagenic effects of HIBs. Their results also indicated that HIBs could lead to clustered DNA damage to the chromosomes. A similar study showed that, compared with Ar ions, C ions induced more single-base substitutions (SBSs) and smaller indels (<100 bp) and demonstrated the different mutational functions of low- and high-LET HIB irradiation [7]. Furthermore, Ichida et al. [14] developed a multiplex and cost-efficient whole-exome sequencing procedure and applied it to reveal the less biased mutation characteristics in the rice genome. Those authors suggested that mutation frequency may be a good indicator for sequencing-based determination of the optimal irradiation conditions for inducing mutations. Using whole-genome resequencing, researchers conducting two recent independent studies compared and characterized mutations induced by CIB irradiation and GRs in rice (*Oryza sativa* L.) [15,16]. Both studies showed that SBSs were the most abundant type of mutation induced by the two mutagens. Furthermore, the researchers found that GR irradiation tended to induce more small mutations than CIB irradiation did, whereas CIB irradiation induced more InDels.

Plants are usually exposed to various DNA-damaging agents because they cannot move like animals. To avoid the accumulation of lesions and preserve their genomic integrity, plants have evolved complex signaling pathways to mediate DNA damage. The DDR network is highly complex and involves multiple pathways and different levels of gene regulation [17,18]. Although recent findings have greatly increased the understanding of mutations induced by heavy-ion beam irradiation and revealed the characteristics of mutations caused by different types of mutagens, most studies have identified heritable mutations only in second- or late-generation plants; however, a large number of mutations cannot be inherited from the first generation of mutant plants. Therefore, information on initial DNA mutations in higher plants remains largely unknown.

Rice (*O. sativa* L.) is one of the most important consumed grains worldwide and feeds more than half of the global population [19]. Among model plant species, rice has a relatively small genome, which makes it especially ideal for mutagenesis research. CIB is a powerful mutagen for plant mutation breeding because of its high LET. Accumulating amounts of evidence have demonstrated that CIB irradiation can cause numerous different types of complex DNA damage, including DSBs, clustered DNA damage, large InDels, and chromosomal rearrangements [20,21]. However, the molecular characteristics and gene expression regulation underlying the initial DNA damage after CIB irradiation are poorly characterized in rice. For this reason, exploring the characteristics and gene expression regulation in response to DNA damage through whole-genome sequencing (WGS) and transcriptomics is a worthwhile subject.

In this study, a time course (48, 96, and 144 h) study of genome-wide mutations and gene expression profiles post-CIB irradiation was performed via whole-genome resequencing and RNA sequencing (RNA-seq) to study the characteristics of initial DNA damage induced by CIB irradiation. We found that both 40 Gy and 80 Gy of CIB irradiation induced point mutations and a high proportion of InDels. Even more interesting is that 40 Gy of CIB irradiation caused more initial mutations than 80 Gy did. We speculate that this may be due to the high dose (80 Gy) of CIB causing a high proportion of nonrepairable damage and leading to cell inhibition and apoptosis. The time course RNA-seq analysis also showed that the number of differentially expressed genes (DEGs) was highest at 48 h post-CIB irradiation and that the expression of several DNA repair-related genes was upregulated. These findings further expand our understanding of the mechanisms underlying initial DNA damage and DSBs induced by CIB irradiation.

## 2. Materials and Methods

### 2.1. Plant Materials and CIB Irradiation

Rice (*O. sativa* L. *japonica* cv. Nipponbare) seeds were exposed to 12 C^6+^ ions at doses of 40 Gy and 80 Gy (LET = 50 keV/µm) generated by the Heavy Ion Research Facility in Lanzhou (HIRFL) at the Institute of Modern Physics, Chinese Academy of Sciences (IMP-CAS).

### 2.2. Whole-Genome Mutation Analysis

After being subjected to 40 and 80 Gy of CIB irradiation, seeds were placed on Petri dishes containing germination paper moistened with 10 mL of sterile water. Fifty seeds were randomly selected from among both the irradiated and non-irradiated seeds at 48, 96, and 144 h after germination, and the embryos were immediately frozen in liquid nitrogen. Genomic DNA was extracted from the collected embryos using the cetyl-trimethylammonium bromide method. DNA libraries were sequenced on the Illumina sequencing platform by Gene Denovo Biotechnology Co., Ltd. (Guangzhou, China). Genomic DNA was randomly sheared into short DNA fragments, end-repaired, A-tailed, and ligated to Illumina paired-end adapters. After purification with Agencourt AMPure XP beads, the DNA fragments approximately 300–400 bp were selected and enriched by PCR. The constructed library was purified and quality-checked, and then sequenced with an Illumina Hiseq X10 PE150 instrument [22]. The data were cleaned and subsequently mapped to the Nipponbare reference genome (IRGSP-1.0) using the Burrows–Wheeler Alignment tool, SAMtools, and Picard-tools. The variants were detected by GATK HaplotypeCaller and annotated by ANNOVAR.

### 2.3. RNA Extraction, Sequencing, and Gene Expression Profile Analysis

Embryos sampled from both the irradiated (40 Gy of CIB irradiation) and non-irradiated seeds at 48, 96, and 144 h after germination (three biological replicates for each treatment) were immediately frozen in liquid nitrogen. Total RNA was extracted using an RNA EasySpin Isolation System (Aidlab Biotech, Beijing, China). The RNA quality was verified using a Bioanalyzer 2100 (Agilent, Santa Clara, CA, USA). The RNA integrity number (RIN) for all the samples was greater than 7, after which the samples were subjected to subsequent analysis. First-strand cDNA was generated using reverse transcriptase and random primers. The cDNA libraries were constructed following procedures described by Liu et al. [23]. The quality of the sequencing data was verified using FastQC (v0.10.1) software, and quality control was confirmed via Cutadapt (v1.9.1). The reads were trimmed and mapped to the IRGSP-1.0 reference genome. After the expression level of each transcript and gene was calculated by using the fragments per kilobase per million mapped fragments (FPKM) method, differential expression analysis was conducted using DESeq2 (v1.6.3) [18]. The DEGs were subjected to Gene Ontology (GO) (http://geneontology.org/, accessed on 6 January 2020) and Kyoto Encyclopedia of Genes and Genomes (KEGG) (http://www.genome.jp/kegg/pathway.htkl, accessed on 6 January 2020) functional enrichment analyses. GO terms with a corrected *p*-value ≤ 0.05 were considered significantly enriched in DEGs. KEGG pathways with a Q-value ≤ 0.05 were considered significantly enriched in DEGs.

## 3. Results

### 3.1. Whole-Genome Resequencing of Irradiated Rice Seeds

To identify CIB irradiation-induced mutations, we obtained a total of 424.69 gigabyte (Gb) of paired-end reads for eight rice samples: two untreated control samples and six CIB-treated samples. For the six CIB-treated samples, the average depth was much greater than 100× (Table 1). The high-quality reads were mapped and covered more than 89% of the Nipponbare genome (IRGSP-1.0) with an average depth of 109× for each sample. For the control samples, the average depth of sequencing are more than 30×. These results suggest that the resequencing data were suitable for subsequent bioinformatics data analysis.

### 3.2. Comprehensive Identification of Mutations Induced by CIB

After treating seeds with a dose of 40 Gy, we identified 447, 1304, and 333 mutations at 48, 96, and 144 h after seed germination, respectively (Table 2). Among these mutations, SBSs were the most abundant type. From 48 to 96 h, the number of both SBSs and InDels increased, but the proportion of InDels decreased. However, the number of mutations decreased dramatically from 96 to 144 h, but the amplitude of the decrease in the SBSs was greater than that in the InDels. Therefore, the number of mutations increased from 48 to 96 h, and the decrease from 96 to 144 h in response to the dose of 40 Gy involved by SBSs. After treating seeds with a dose of 80 Gy, we identified 202, 828 and 1307 mutations at 48, 96, and 144 h after seed germination, respectively. The number of mutations progressively increased from 48 to 144 h, especially the number and proportion of SBSs. While the number of InDels also increased, their proportion significantly decreased from 48 h (43.07%) to 144 h (9.49%). In general, whether 40 Gy or 80 Gy was applied, the proportion of InDels exceeded 40% in the initial stage (48 h) after CIB irradiation in rice. Taken together, these results indicate that CIB irradiation can induce not only a large amount of SBSs but also many InDels.

### 3.3. Mutation Frequency and Clustered DNA Damage Induced by CIB

**Figure 1:**
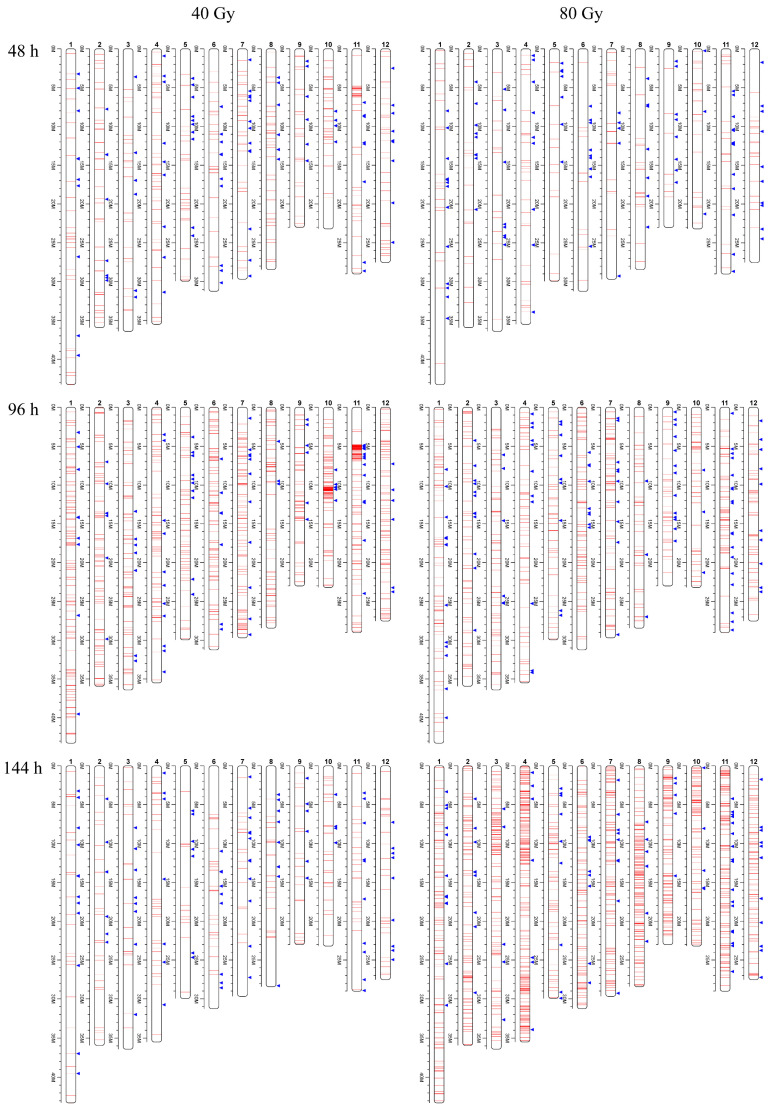
Distribution of SBSs and small InDels induced by CIB irradiation across chromosomes. The red lines indicate SBSs and the blue lines indicate InDels.

To study the dynamic differences in mutations between the two irradiation doses at different time points, we first calculated the mutation frequency in response to the two irradiation doses at different time points after germination. In general, regardless of whether 40 Gy or 80 Gy of CIB irradiation was applied, we observed an increase in mutation frequency from 48 to 96 h (Figure 1). However, the mutation frequency decreased in response to 40 Gy of CIB irradiation at 144 h after germination, whereas it tended to continuously increase in response to 80 Gy of CIB irradiation. Moreover, a high mutation frequency was found on chromosome 10 (Chr10) and chromosome 11 (Chr11) in response to 40 Gy of CIB irradiation at 96 h after germination (Figure 1), and their mutation frequencies were 0.8–2.2 times higher than the average level throughout the whole genome. We subsequently measured and visualized the distribution of mutations across the chromosomes. A total of 177 mutations were detected in the 5–6 Mb region of Chr11 and 86 and 54 mutations were detected in its adjacent regions (4–5 and 6–7 Mb). Although these mutations encompassed only a 10% region of Chr11, they constituted 87.09% of all the mutations of Chr11. By visualizing these variations, we identified these mutations as composing clustered DNA lesions caused by CIB irradiation. Notably, the mutation frequency was not proportional to the length of the chromosome for either dose of CIB irradiation. For example, Chr10 is relatively short, but its mutation frequency was higher than the average level across the whole genome.

### 3.4. Distribution and Rates of Mutations Induced by CIB Irradiation

**Figure 2:**
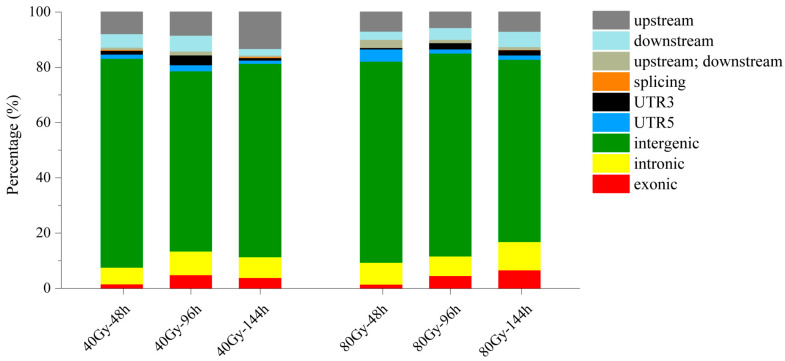
Locations of mutations in the genome induced by CIB irradiation.

Regardless of whether 40 Gy or 80 Gy of CIB irradiation was applied, mutations were induced predominantly in intergenic regions (65.18–75.66%), followed by upstream and downstream regions (11.11–16.22%), intronic regions (6.04–10.25%), UTR3 and UTR5 (2.10–5.75%), and exonic regions (1.49–6.66%) (Figure 2). Generally, mutations induced in an exonic gene region may have a high impact on gene function. At 144 h after seed germination, 13 and 87 mutations were induced in the exonic region in response to 40 Gy and 80 Gy, respectively. At the initial stage (48 h post-irradiation), although 40 Gy of CIB irradiation induced more mutations than 80 Gy of CIB irradiation did, most occurred in the intergenic regions and were less frequent in the exonic regions (both of which had fewer than eight). However, with cell proliferation and DNA replication, 80 Gy of CIB irradiation induced exonic mutations 6.7 times more frequently than 40 Gy of CIB irradiation did. As a result, 80 Gy of CIB irradiation may have higher mutagenic efficiency than 40 Gy of CIB irradiation in practical breeding.

### 3.5. Characteristics of SBSs and InDels Induced by CIB Irradiation

**Figure 3:**
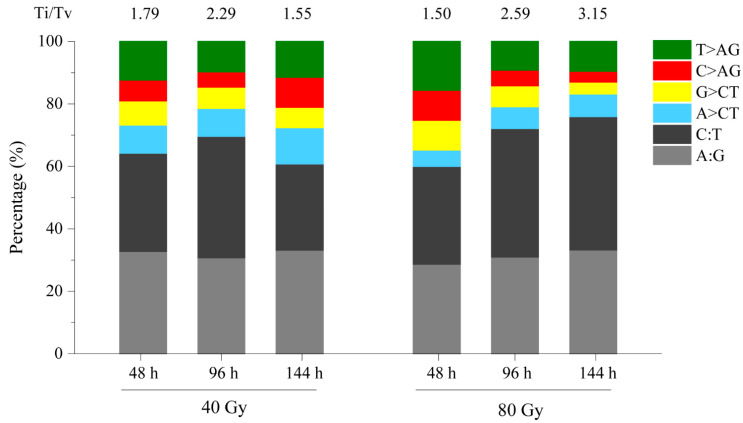
Ratio of transitions to transversions (Ti/Tv) induced by CIB irradiation. A:G and C:T are transitions. A > C/T, G > C/T, C > A/G, and T > A/G are transversions.

Among the mutations induced by CIB irradiation, SBSs were the most abundant and were induced by both 40 Gy and 80 Gy CIBs. We found that C:T and A:G transitions were the two major types of mutations induced in response to both doses of CIB irradiation, and their proportion (60.00–75.91%) was significantly higher than that of the other types of mutations (Figure 3). From 48 to 96 h, the proportion of C:T and A:G transitions obviously increased in response to both doses of CIB irradiation, while it decreased in response to 40 Gy and increased in response to 80 Gy from 96 to 144 h. At the initial stage (48 h), we identified 98 (32.78%) and 33 (28.70%) A:G transitions and 94 (31.44%) and 36 C:T (31.30%) transitions under the 40 Gy and 80 Gy CIB irradiation, respectively. However, at 144 h, the proportion of A:G transitions was similar for both doses of CIB irradiation, while the proportion of C:T transitions in the 80 Gy group was significantly higher than that in the 40 Gy group. As a result, the Ti/Tv ratio in response to 80 Gy of CIB irradiation was 2 times greater than that in response to 40 Gy of CIB irradiation at 144 h.

**Figure 4:**
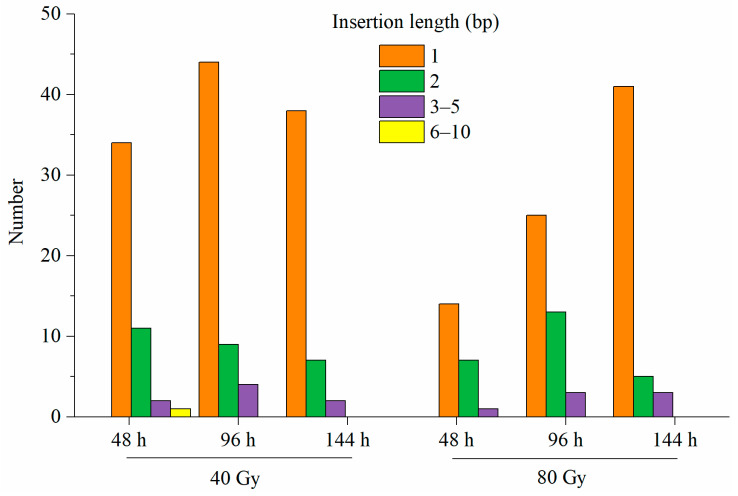
Frequencies and sizes of insertions identified after CIB irradiation.

**Figure 5:**
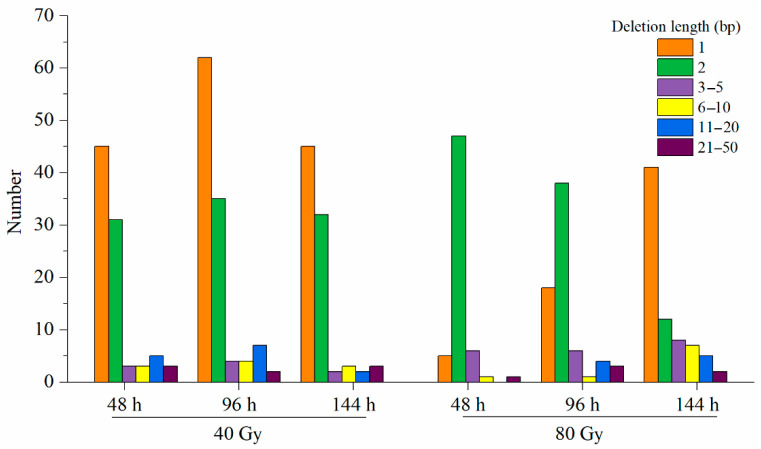
Frequencies and sizes of deletions identified after CIB irradiation.

Among insertions, single-base ones were the most frequent in response to both doses (40 and 80 Gy) of CIB irradiation. The size of the insertions varied from 1 to 10 bp, but the majority were 1–5 bp (Figure 4). With respect to the number of insertions, there was no significant difference between the two doses of CIB irradiation, and the insertion sizes were similar. However, with respect to deletions, 40 Gy of CIB irradiation seemed to induce more deletions than 80 Gy CIB irradiation did. In addition, single-base deletions and 2 bp deletions were the most frequent in response to 40 and 80 Gy CIB irradiation, respectively. Overall, regardless of 40 or 80 Gy of CIB irradiation was applied, short InDels (<2 bp) were more frequent than long InDels were (Figure 5). Interestingly, at the initial stage (48 h), 40 Gy of CIB irradiation induced long InDels (>5 bp) more frequently than 80 Gy of CIB irradiation did, but at the later stage (144 h), we identified more long InDels (>5 bp) in response to 80 Gy of CIB irradiation than in response to 40 Gy of CIB irradiation.

### 3.6. Genome-Wide Expression Changes after CIB Irradiation

**Figure 6:**
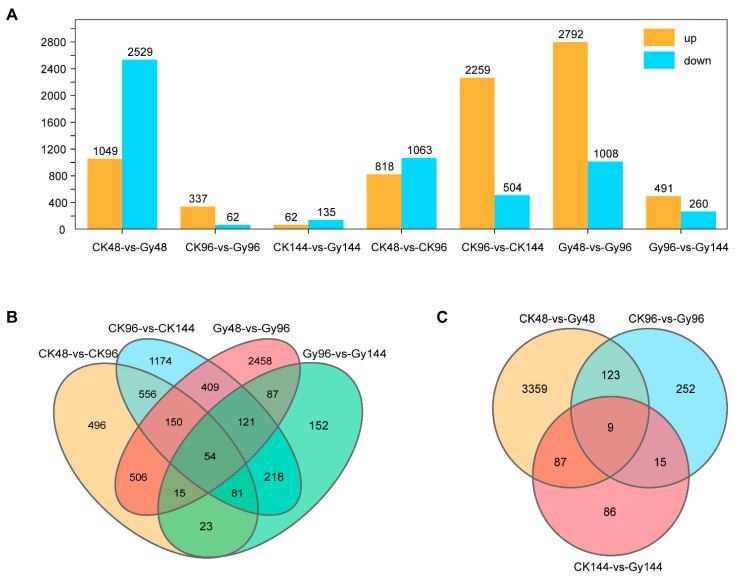
Analysis of differentially expressed genes (DEGs) between control group (CK) and irradiated samples (Gy). (**A**) The number of DEGs between control group (CK) and irradiated samples (Gy) at three time points after 40 Gy CIB. (**B**) Venn diagram of DEGs in the same treatment at different times. (**C**) Venn diagram of DEGs at three different times for the CK vs. Gy transcriptome comparison.si.

To further characterize the rice genes expressed in response to CIB irradiation, we treated imbibed *O. sativa* seeds with 40 Gy of CIB irradiation; sampled the seeds at 48, 96, and 144 h after germination; and conducted RNA-seq analysis together with a control. In total, 18 samples were subjected to RNA-seq, resulting in 4.8-Gb 150-bp paired-end reads per sample on average (Supplementary Table S1). Genes whose expression was significantly up- or downregulated in accordance with a |log2(fold change)| ≥ 2 and *p* < 0.05 were considered differentially expressed. When the genes in the treatment group were compared with those in control group, there were 1881 DEGs (818 and 1063 whose expression was upregulated and downregulated, respectively) at 48 h vs. 96 h and 2763 DEGs (2259 and 504 whose expression was upregulated and downregulated, respectively) at 96 h vs. 144 h. For the 40 Gy CIB treatment group, there were 3800 DEGs (2792 and 1008 whose expression was upregulated and downregulated, respectively) at 48 h vs. 96 h and 751 DEGs (491 and 260 whose expression was upregulated and downregulated, respectively) at 96 h vs. 144 h (Figure 6A). When the data at the three time points (48, 96 and 144 h) were compared, there were 3578, 399, and 197 DEGs between the 40 Gy CIB treatment group and control group, respectively (Figure 6A). At the initial stage (48 h), there were 1049 genes whose expression was upregulated and 2529 genes whose expression was downregulated in the treatment group. However, at 96 and 144 h, a small number of DEGs were detected. This may be because plants are more inclined to initiate damage repair mechanisms early upon experiencing DNA damage. Venn diagram analysis showed a large number of DEGs were overlapped between the same treatment at different times, indicating that these genes were related to the growth and development of rice. However, only nine overlapped DEGs were identified at three time points for the CK vs. Gy transcriptome comparison.si., indicating that these genes were steadily differentially expressed between the CK and Gy.

### 3.7. Time Course Enrichment Analysis of GO Terms and KEGG Pathways

**Figure 7:**
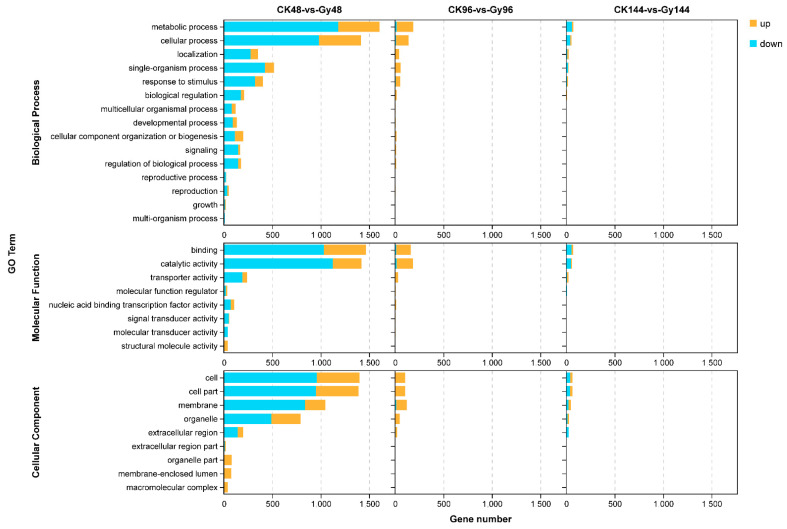
GO analysis of DEGs whose expression was up-and downregulated in the CK vs. Gy comparison. CK, control samples; Gy, samples treated with 40 Gy of CIB irradiation.

To further characterize the genes expressed in response to CIB irradiation, we performed GO enrichment and KEGG pathway analyses of the DEGs. The DEGs in the 40 Gy of CIB irradiation group and the control group at the three time points (48, 96, and 144 h) were classified into three main GO categories: biological processes (BPs), cellular components, and molecular functions (MFs) (Figure 7). For the BP category, the GO terms that were most enriched were “metabolic process”, “cellular process”, “single-organism process”, and “response to stimulus”. For the MF category, the top three functional terms involving most DEGs were “binding”, “catalytic activity”, and “transporter activity”. For the BP category, “cell”, “cell part”, “membrane”, and “organelle” were the most frequently assigned GO terms.

**Figure 8:**
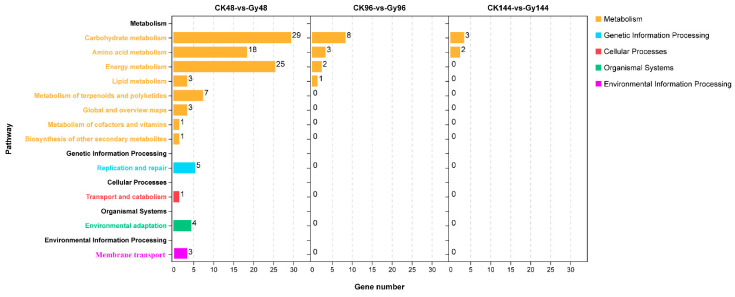
KEGG pathway enrichment of DEGs whose expression was up- and downregulated in the CK vs. Gy comparison. CK, control samples; Gy, samples treated with 40 Gy of CIB irradiation.

To further explore the biological functions of the DEGs, an enrichment analysis based on the content within the KEGG pathway database was performed. The top 12 KEGG pathways involving most DEGs are listed in Figure 8. At 48 h, the DEGs were enriched mostly in terms such as “carbohydrate metabolism”, “energy metabolism”, “amino acid metabolism”, “carbon metabolism”, “metabolism of terpenoids and polyketides”, and “replication and repair”, whereas at 96 and 144 h, the DEGs were enriched mostly in “carbohydrate metabolism” and “amino acid metabolism”. Of the three time points, only at 48 h were different enrichment pathways revealed, such as “metabolism of terpenoids and polyketides” and “replication and repair”.

### 3.8. Variation in Transcript Levels of Damage/Repair-Related Genes Following CIB Irradiation

**Figure 9:**
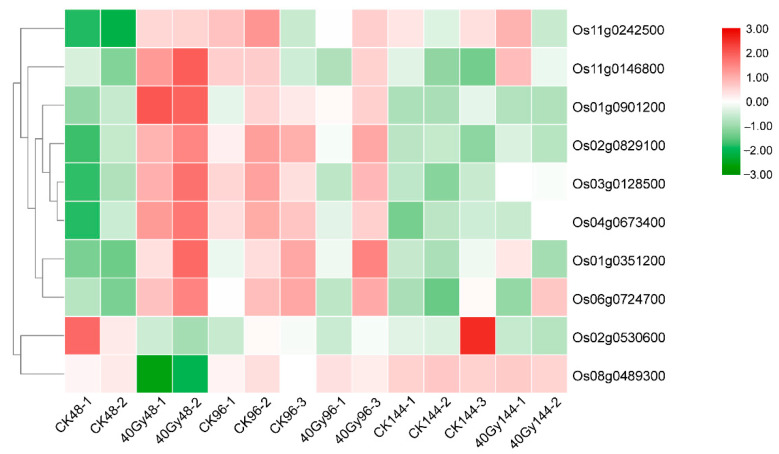
Heat map of the expression trends of 10 DNA repair-related genes.

Since a larger number of DEGs were identified at 48 h, many fewer DEGs were detected at 96 and 144 h. Additionally, KEGG enrichment analysis showed that the “replication and repair” pathway was significantly enriched only at 48 h. These results suggest that the expression of a number of DNA repair-related genes and pathways may be induced rapidly (48 h or earlier) in response to DNA damage. Therefore, we focused on the expression of genes related to DNA repair after exposure to CIB irradiation for 48 h. Of the known DNA repair-related genes in rice, ten genes involved in the DNA repair pathway increased in expression after exposure to CIB irradiation for 48 h (Table 3 and Figure 9). Among them, *Os01g0901200*, an orthologue of *AT2G19490* in *Arabidopsis*, encodes a recA protein involved in recombination-dependent repair and plays an important role in the maintenance of organelle genome integrity [24,25]. The expression of *Os01g0901200* was upregulated at 48 h after germination in response to 40 Gy of CIB irradiation. Two DSB repair-related genes, *Os01g0351200* and *Os02g0829100*, which are orthologues of *PARP2* (*AT4G02390*) and *PARP3* (*AT5G22470*), respectively, in *Arabidopsis* were detected as genes whose upregulated expression depended on CIB irradiation in this study. Previous studies have shown that PARP family genes not only are involved in Ku-independent non-homologous end-joining (NHEJ) (alternative (A)-NHEJ) in animals but also play a role in the A-NHEJ pathway in plants [26,27]. Additionally, another gene, *Os04g0673400*, whose expression was upregulated encodes a uracil-DNA glycosylase, and its *Arabidopsis* homologous gene *AT3G18630* has been reported to be involved in a base-excision DNA repair pathway in mitochondria [28,29,30]. *Os06g0724700* is also a gene whose expression was upregulated at 48 h after germination in response to 40 Gy of CIB irradiation; this gene encodes a phosphatidylinositol kinase and FAT domain-containing protein, and its *Arabidopsis* homologous gene *AT5G40820* encodes an *Arabidopsis* orthologue of the ATR protein kinase that is both involved in a wide range of responses to DNA damage and plays a central role in cell cycle regulation [31,32].

## 4. Discussion

As mutation breeding increases and breeding programs in space raise concerns, it is important to study the basic mechanisms underlying the biological effects and DNA damage in response to heavy ions. To study the dynamic changes in initial DNA damage and gene expression in response to CIB irradiation, we characterized the mutations and gene expression profiles in rice after CIB irradiation by a WGS and an RNA-seq time course analysis. Overall, under 40 Gy or 80 Gy, single-base substitutions (SBSs) were the main mutation types induced by CIB irradiation. Moreover, CIB irradiation also induced a number of InDels, and short ones (<5 bp) were more prevalent than large ones, which is in accordance with the findings of previous studies [15,16]. Among CIB irradiation-induced SBSs, we detected a bias towards C:T and A:G transitions and observed a higher overall frequency of CIB irradiation-induced transversions versus transitions (Figure 3). This tendency seems common in response to heavy-ion beam irradiation, whether high or low LTE, Ar ions, or C ions are used, and is unrelated to the irradiation dose [7,13]. These findings imply that the bias of mutations might be caused by similar mechanisms. Moreover, we also observed some obviously different characteristics of CIB irradiation after different time points. The number and frequency of mutations induced by 40 Gy of CIB irradiation tended to increase first but then decrease, while they continuously increased in response to 80 Gy of CIB irradiation over time. However, even more amazing is that we detected more mutations under the 40 Gy dose than under the 80 Gy dose at 48 h after germination. However, a much higher number of mutations was observed in response to the 80 Gy dose than in response to the 40 Gy dose at 144 h after germination. CIB irradiation can lead to severe damage (such as DSBs); subsequently, it induces cell cycle arrest to allow time for repair and eventually results in cell apoptosis. A previous study showed that the apoptosis of human lymphocytes induced by HIB irradiation occurs early. The apoptosis mechanism was initiated in cells within 12 h of exposure, and the number of apoptotic cells increased. The apoptosis rate peaked at 24 h; afterwards, with increasing time, the apoptosis rate gradually decreased and returned to the control level by approximately 30 days [18]. In fact, the apoptosis rate increases with increasing radiation dose. Therefore, we believe that fewer mutations were detected in response to 80 Gy of CIB irradiation than to 40 Gy of CIB irradiation at 48 h because of severe genome damage caused by the former, leading to cell apoptosis. Most of the severely damaged cells underwent programmed cell death, so the number of cells that could be used for sequencing was extremely small.

Baselet et al. [33] used microarrays and focus quantification to compare the different effects on endothelial cells irradiated with different doses of individual X-rays measured after various post-irradiation (repair) times. The results showed that the focus numbers were greatest at 30 min to 1 h after irradiation, followed by an almost complete decline at 24 h. Compared to those irradiated with the low dose, endothelial cells irradiated with a single X-ray dose of 2 Gy had higher cellular proliferation at day 7 post-irradiation. The researchers suggested that the increased proliferation was probably due to the absence of contact inhibition caused by cell death and the longer and stronger G1 arrest induced at day 1. This evidence and these findings may explain why 80 Gy of CIB irradiation induced fewer mutations than 40 Gy of CIB irradiation did at 48 h post-irradiation, and the mutation numbers continued to increase and were higher than those in response to 40 Gy at 144 h post-irradiation.

Clustered DNA damage is a specific type of DNA damage induced by ionizing radiation [20], especially HIB irradiation. This same induction characteristic of CIB irradiation is quite different from those of chemical mutagens. High-LET irradiation (such as protons, fast neutrons, and heavy-ion beams) occurs along the particle track, resulting in clusters of ionization events and induced mutations of clustered lesions [18,21]. However, the principle of chemical mutagenesis is that the structure of DNA has been replaced by several active groups of chemical substances, so the mutation sites of chemical mutagenesis are limited. In principle, the number of mutations inducible in a genome is linearly correlated with irradiation dose (Ichida et al., 2018). A previous study showed that clustered DNA damage yields decreased with increased LET. However, the LET in our experimental design remained unchanged. Interestingly, an unexpected result of our study is that we did not observe obvious DNA cluster damage caused by 80 Gy of CIB irradiation. Compared with a low radiation dose, a high radiation dose is more intensive and more likely to cause clustered DNA damage [34]. However, excessive unrepairable damage can lead to cell inhibition and apoptosis. Unrepairable damage to the essential genes of plants is especially more likely to result in apoptosis to maintain the stability of the genome. Therefore, we believe the reason for the lower initial mutations and DNA cluster damage observed in response to 80 Gy of CIB irradiation compared to 40 Gy of CIB irradiation is that a high portion of nonrepairable damage occurred in response to the 80 Gy of CIB irradiation, which resulted in fewer cells available for resequencing.

We also characterized the gene expression profiles of rice seeds after CIB irradiation. The largest number of genes whose expression was up-/downregulated was detected at 48 h post-irradiation (Figure 6). Moreover, the GO terms “metabolic process”, “cellular process”, “single-organism process”, and “response to stimulus” within the BP ontology and “binding”, “catalytic activity”, and “transporter activity” in the MF ontology were enriched at 48 h post-irradiation. These GO terms were also found to be enriched in another gene expression profiling study involving artificial mutations induced by ethylmethanesulfonate in eggplant (*Solanum melongena* L.) [6] and HIB irradiation of rice [35]. Otherwise, the KEGG pathways “metabolism of terpenoids and polyketides”, “environmental adaptation”, and “replication and repair” were enriched only at 48 h post-irradiation. Taken together, these data suggest that this time point was ideal for profiling changes in gene expression after HIB irradiation in this study. As expected, the expression of some known DNA repair-related genes was strongly upregulated at 48 h post-irradiation (Figure 9). Therefore, we focused on the DEGs at this time point, especially DNA repair-related genes. CIB irradiation causes multiple types of DNA damage, including SSBs and DNA DSBs. Of these, DSBs pose a major threat that affects cellular fate because, if these breaks are not repaired before cell division, they may lead to cell death or carcinogenesis. Upon detection of a DSB, many repair pathways may be activated, including the classic non-homologous end-joining (cNHEJ) pathway and the recently discovered aNHEJ sub-pathway. The expression of two DSB repair-related genes, *Os01g0351200* and *Os02g0829100*, which are orthologues of *PARP2* (*AT4G02390*) and *PARP3* (*AT5G22470*) in *Arabidopsis*, was upregulated genes at 48 h post-irradiation in this study (Figure 9). Poly(ADP-ribosyl)ation (PARylation) is an important post-translational modification involved in the regulation of DNA repair in multicellular organisms. In higher eukaryotes, NHEJ is considered the main pathway of DSB repair. A previous study showed that *PARP1* and *PARP2* are involved in the B-NHEJ repair pathway in *Arabidopsis* [36,37]. Thus, some PARP family genes might also play an important role in repair pathways in rice. We assume that the high expression of *Os01g0351200* and *Os02g0829100* observed at 48 h post-irradiation is needed to repair the high-LET-induced DNA lesions via A-NHEJ. Although many characteristics of mutations caused by CIB irradiation have been revealed through WGS and RNA-seq analyses [7,35], most studies identified these mutations via whole-genome shotgun sequencing. However, heavy ions can also generate numerous structural variations and chromosomal rearrangement events. Unfortunately, second-generation Illumina sequencing has low reliability in terms of the detection of large-segment structural variation and chromosomal rearrangements. Therefore, with the development of third-generation sequencing techniques, which benefit from long-read sequencing platforms, we can use PacBio Single Molecule Real-Time (SMRT) sequencing technology to reveal more characteristics of structural variation and chromosomal rearrangements induced by HIB irradiation in the future. 

## 5. Conclusions

In conclusion, we found that SBSs was the most frequent type of mutation induced by CIB. Notably, more mutations have been detected with 40 Gy carbon ion beam(CIB) than 80 Gy at initial stage, this may due to the 80 Gy carbon ion beams caused serious damage and resulting the number of cells that can be used for sequencing is extremely small. Furthermore, we also observed closely spaced DNA lesions forming a cluster DNA damage on some chromosome regions after 96 h irradiation of 40 Gy CIB. RNA-seq analysis at three time points showed that the number of differentially expressed genes (DEGs) was highest at 48 h post-irradiation. Kyoto Encyclopaedia of Genes and Genomes (KEGG) pathway analysis of DEGs showed that “replication and repair” pathway was specifically enriched 48 h post-irradiation. Among these DEGs, ten DNA repair-related genes were up-regulated at 48 h post-irradiation. These results indicated that the DNA damage response (DDR) and the mechanism of DNA repair are tends to quickstart within an initial stage (48 h) after irradiation. Our study not only provides valuable information for the damage and repair characteristics by CIB irradiation, but also supply a helpful reference to the selection of key time points in further researches.

## Figures and Tables

**Table 1:** Re-sequencing and quality control information of the control and treated samples.

Sample	Clean Data (bp)	HQ Clean Data (bp)	Total Reads	Total Unmapped Reads	Total Mapped Reads	Average Depth
CK-40Gy	1.2 × 10^10^	10,699,457,320	71,555,678	9,994,707 (13.97%)	61,560,971 (86.03%)	32.16×
40Gy-48h	6.539 × 10^10^	60,338,857,661	403,481,808	102,161,779 (25.32%)	301,320,029 (74.68%)	175.20×
40Gy-96h	5.475 × 10^10^	51,836,646,543	346,316,164	116,046,170 (33.51%)	230,269,994 (66.49%)	146.69×
40Gy-144h	5.209 × 10^10^	51,735,401,305	345,632,662	68,450,817 (19.80%)	277,181,845 (80.20%)	139.55×
CK80Gy	1.281 × 10^10^	12,669,338,558	84,647,388	5,273,573 (6.23%)	79,373,815 (93.77%)	34.32×
80Gy48h	4.043 × 10^10^	39,926,015,703	266,742,060	32,349,666 (12.13%)	234,392,394 (87.87%)	108.33×
80Gy96h	4.081 × 10^10^	40,346,411,461	269,666,882	19,379,819 (7.19%)	250,287,063 (92.81%)	109.34×
80Gy144h	4.807 × 10^10^	47,692,554,563	318,715,200	24,827,815 (7.79%)	293,887,385 (92.21%)	128.79×

**Table 2:** Number of SBSs and InDels induced by different doses of CIB irradiation at different time points after irradiation.

Treatment	Time	Total	Number of Mutations			Proportion of Each Mutation (%)		
			SBSs	Insertions	Deletions	SBSs	Insertions	Deletions
40 Gy	48 h	447	299	94	54	66.89%	21.03%	12.08%
	96 h	1304	1133	114	57	86.89%	8.74%	4.37%
	144 h	333	199	87	47	59.76%	26.13%	14.11%
80 Gy	48 h	202	115	57	30	56.93%	28.22%	14.85%
	96 h	828	717	70	41	86.59%	8.45%	4.95%
	144 h	1307	1183	75	49	90.51%	5.74%	3.75%

**Table 3:** Information concerning ten known DNA repair-related genes.

Os ID	Gene Description	AT ID	GO Biological Processes
*Os01g0351200*	Poly synthetase 2-A	*AT4G02390*	DNA ADP-ribosylation; double-stranded break repair via non-homologous end joining; protein poly-ADP-ribosylation
*Os01g0901200*	RecA protein	*AT2G19490*	DNA repair
*Os02g0829100*	Poly synthetase 3	*AT5G22470*	Double-stranded break repair; protein poly-ADP-ribosylation
*Os03g0128500*	POLD2-Putative DNA polymerase delta complex subunit	*AT2G42120*	DNA strand elongation involving DNA replication
*Os04g0673400*	Uracil-DNA glycosylase	*AT3G18630*	Base-excision repair; AP site formation via deaminated base removal
*Os06g0724700*	Phosphatidylinositol kinase and FAT domain-containing protein	*AT5G40820*	DNA repair, double-stranded break repair via non-homologous end joining, response to γ radiation
*Os11g0146800*	*OsDMC1B*		
*Os11g0242500*	Cyclin-dependent kinase		
*Os02g0530600*	Polysynthetase 3	*AT5G22470*	Double-stranded break repair, protein poly-ADP-ribosylation
*Os08g0489300*	Methyladenine glycosylase	*AT1G13635*	Base-excision repair

## Data Availability

The RNA-seq data used in these studies are available from https://www.ncbi.nlm.nih.gov/sra/?term=SRP323483, accessed on 10 June 2021.

## Supplementary Materials

The following are available online at https://www.mdpi.com/article/10.3390/genes12091391/s1, Table S1: Quality information of RNA-seq data.

## References

[1] Saeb A.T.M., Al-Naqeb D. (2016). The Impact of Evolutionary Driving Forces on Human Complex Diseases: A Population Genetics Approach. Scientifica.

[2] Ossowski S., Schneeberger K., Lucas-Lledó J.I., Warthmann N., Clark R.M., Shaw R.G., Weigel D., Lynch M. (2010). The Rate and Molecular Spectrum of Spontaneous Mutations in *Arabidopsis thaliana*. Science.

[3] Shirley B.W., Hanley S., Goodman H.M. (1992). Effects of ionizing radiation on a plant genome: Analysis of two *Arabidopsis transparent* testa mutations. Plant Cell.

[4] Morita R., Kusaba M., Iida S., Yamaguchi H., Nishio T., Nishimura M. (2009). Molecular characterization of mutations induced by γ irradiation in rice. Genes Genet. Syst..

[5] Belfield E.J., Gan X., Mithani A., Brown C., Jiang C., Franklin K., Alvey E., Wibowo A., Jung M., Bailey K. (2012). Genome-wide analysis of mutations in mutant lineages selected following fast-neutron irradiation mutagenesis of *Arabidopsis thaliana*. Genome Res..

[6] Xiao X.-o., Lin W., Li K., Feng X., Jin H., Zou H. (2019). Genome-Wide Analysis of Artificial Mutations Induced by Ethyl Methanesulfonate in the Eggplant (*Solanum melongena* L.). Genes.

[7] Kazama Y., Ishii K., Hirano T., Wakana T., Yamada M., Ohbu S., Abe T. (2017). Different mutational function of low- and high-linear energy transfer heavy-ion irradiation demonstrated by whole-genome resequencing of *Arabidopsis* mutants. Plant J..

[8] Tanaka A., Shikazono N., Hase Y. (2010). Studies on Biological Effects of Ion Beams on Lethality, Molecular Nature of Mutation, Mutation Rate, and Spectrum of Mutation Phenotype for Mutation Breeding in Higher Plants. J. Radiat. Res..

[9] Abe T., Kazama Y., Hirano T. (2015). Ion Beam Breeding and Gene Discovery for Function Analyses Using Mutants. Nucl. Phys. News.

[10] Du Y., Luo S., Li X., Yang J., Cui T., Li W., Yu L., Feng H., Chen Y., Mu J. (2017). Identification of Substitutions and Small Insertion-Deletions Induced by Carbon-Ion Beam Irradiation in *Arabidopsis thaliana*. Front. Plant Sci..

[11] Shirasawa K., Hirakawa H., Nunome T., Tabata S., Isobe S. (2016). Genome-wide survey of artificial mutations induced by ethyl methanesulfonate and γ rays in tomato. Plant Biotechnol. J..

[12] Li S., Zheng Y.-C., Cui H.-R., Fu H.-W., Shu Q.-Y., Huang J.-Z. (2016). Frequency and type of inheritable mutations induced by γ rays in rice as revealed by whole genome sequencing. J. Zhejiang Univ. Sci. B.

[13] Hirano T., Kazama Y., Ishii K., Ohbu S., Shirakawa Y., Abe T. (2015). Comprehensive identification of mutations induced by heavy-ion beam irradiation in *Arabidopsis thaliana*. Plant J..

[14] Ichida H., Morita R., Shirakawa Y., Hayashi Y., Abe T. (2019). Targeted exome sequencing of unselected heavy-ion beam-irradiated populations reveals less-biased mutation characteristics in the rice genome. Plant J..

[15] Yang G., Luo W., Zhang J., Yan X., Du Y., Zhou L., Li W., Wang H., Chen Z., Guo T. (2019). Genome-Wide Comparisons of Mutations Induced by Carbon-Ion Beam and γ-Rays Irradiation in Rice via Resequencing Multiple Mutants. Front. Plant Sci..

[16] Li F., Shimizu A., Nishio T., Tsutsumi N., Kato H. (2019). Comparison and Characterization of Mutations Induced by γ-Ray and Carbon-Ion Irradiation in Rice (*Oryza sativa* L.) Using Whole-Genome Resequencing. G3 Genes Genomes Genet..

[17] Mannuss A., Trapp O., Puchta H. (2012). Gene regulation in response to DNA damage. Biochim. Biophys. Acta Gene Regul. Mech..

[18] Missirian V., Conklin P.A., Culligan K.M., Huefner N.D., Britt A.B. (2014). High atomic weight, high-energy radiation (HZE) induces transcriptional responses shared with conventional stresses in addition to a core “DSB” response specific to clastogenic treatments. Front. Plant Sci..

[19] Ochoa M., Tierra W., Tupuna-Yerovi D.S., Guanoluisa D., Otero X.L., Ruales J. (2020). Assessment of cadmium and lead contamination in rice farming soils and rice (*Oryza sativa* L.) from Guayas province in Ecuador. Environ. Pollut..

[20] Hada M., Georgakilas A.G. (2008). Formation of Clustered DNA Damage after High-LET Irradiation: A Review. J. Radiat. Res..

[21] Tokuyama Y., Furusawa Y., Ide H., Yasui A., Terato H. (2015). Role of isolated and clustered DNA damage and the post-irradiating repair process in the effects of heavy ion beam irradiation. J. Radiat. Res..

[22] Ma Q., Wang J., Qi J., Peng D., Guan B., Zhang J., Li Z., Zhang H., Li T., Shi Y. (2021). Increased chromosomal instability characterizes metastatic renal cell carcinoma. Transl. Oncol..

[23] Liu F., Xie L., Yao Z., Zhou Y., Zhou W., Wang J., Sun Y., Gong C. (2019). Caragana korshinskii phenylalanine ammonialyase is up-regulated in the phenylpropanoid biosynthesis pathway in response to drought stress. Biotechnol. Biotechnol. Equip..

[24] Odahara M., Kishita Y., Sekine Y. (2017). MSH1 maintains organelle genome stability and genetically interacts with RECA and RECG in the moss *Physcomitrella* patens. Plant J..

[25] Rowan B.A., Oldenburg D.J., Bendich A.J. (2010). RecA maintains the integrity of chloroplast DNA molecules in Arabidopsis. J. Exp. Bot..

[26] Manova V., Gruszka D. (2015). DNA damage and repair in plants—From models to crops. Front. Plant Sci..

[27] Gu Z., Pan W., Chen W., Lian Q., Wu Q., Lv Z., Cheng X., Ge X. (2019). New perspectives on the plant PARP family: Arabidopsis PARP3 is inactive, and PARP1 exhibits predominant poly (ADP-ribose) polymerase activity in response to DNA damage. BMC Plant Biol..

[28] Wynn E., Purfeerst E., Christensen A. (2020). Mitochondrial DNA Repair in an Arabidopsis thaliana Uracil N-Glycosylase Mutant. Plants.

[29] Boesch P., Ibrahim N., Paulus F., Cosset A., Tarasenko V., Dietrich A. (2009). Plant mitochondria possess a short-patch base excision DNA repair pathway. Nucleic Acids Res..

[30] Elo A., Lyznik A., Gonzalez D.O., Kachman S.D., Mackenzie S.A. (2003). Nuclear Genes That Encode Mitochondrial Proteins for DNA and RNA Metabolism Are Clustered in the Arabidopsis Genome. Plant Cell.

[31] Feng W., Hale C.J., Over R.S., Cokus S.J., Jacobsen S.E., Michaels S.D. (2017). Large-scale heterochromatin remodeling linked to overreplication-associated DNA damage. Proc. Natl. Acad. Sci. USA.

[32] Mahapatra K., Roy S. (2020). An insight into the mechanism of DNA damage response in plants- role of SUPPRESSOR OF γ RESPONSE 1: An overview. Mutat. Res. Mol. Mech. Mutagen..

[33] Baselet B., Belmans N., Coninx E., Lowe D., Janssen A., Michaux A., Tabury K., Raj K., Quintens R., Benotmane M.A. (2017). Functional Gene Analysis Reveals Cell Cycle Changes and Inflammation in Endothelial Cells Irradiated with a Single X-ray Dose. Front. Pharmacol..

[34] Georgakilas A.G., O’Neill P., Stewart R.D. (2013). Induction and Repair of Clustered DNA Lesions: What Do We Know So Far?. Radiat. Res..

[35] Ishii K., Kazama Y., Morita R., Hirano T., Ikeda T., Usuda S., Hayashi Y., Ohbu S., Motoyama R., Nagamura Y. (2016). Linear Energy Transfer-Dependent Change in Rice Gene Expression Profile after Heavy-Ion Beam Irradiation. PLoS ONE.

[36] Jia Q., Dulk-Ras A.D., Shen H., Hooykaas P.J.J., de Pater S. (2013). Poly(ADP-ribose)polymerases are involved in microhomology mediated back-up non-homologous end joining in Arabidopsis thaliana. Plant. Mol. Biol..

[37] Song J., Keppler B.D., Wise R.R., Bent A.F. (2015). PARP2 Is the Predominant Poly(ADP-Ribose) Polymerase in Arabidopsis DNA Damage and Immune Responses. PLoS Genet..

